# Supplementary material for: PARP Inhibitors as Monotherapy in Daily Practice for Advanced Prostate Cancers
Source: J Clin Med. 2022 Mar 21;11(6):1734. doi: 10.3390/jcm11061734 (PMC8952857; doi:10.3390/jcm11061734)
Supplement: Supplementary file 1 [file jcm-11-01734-s001.zip › jcm-1605967-supplementary.pdf]

Members of the ccAFU: Charles Dariane, Eric Barret, Jean-Baptiste Beauval, Laurent Brureau, Gilles Créhange, Gaëlle Fiard, Gaëlle Fromont, Mathieu Gauthé, Alain Ruffion, Raphaële Renard-Penna, Romain Mathieu, Paul Sargos, Morgan Rouprêt, Guillaume Ploussard, Guilhem Roubaud
